# Editorial: An Open Book: What and How Young Children Learn from Picture and Story Books

**DOI:** 10.3389/fpsyg.2015.01719

**Published:** 2015-11-10

**Authors:** Jessica S. Horst, Carmel Houston-Price

**Affiliations:** ^1^School of Psychology, University of SussexBrighton, UK; ^2^School of Psychology and Clinical Language Sciences, University of Reading MalaysiaNusajaya, Malaysia

**Keywords:** storybook attributes, word learning, stereotypes, anthropomorphism, cross-cultural comparisons

Looking at and listening to picture and story books is a ubiquitous activity, frequently enjoyed by many young children and their parents. Well before children can read for themselves they are able to learn from books. Looking at and listening to books increases children's general knowledge, understanding about the world, and promotes language acquisition. This collection of papers demonstrates the breadth of information pre-reading children learn from books and increases our understanding of the social and cognitive mechanisms that support this learning. Our hope is that this Research Topic/eBook will be useful for researchers as well as educational practitioners and parents who are interested in optimizing children's learning.

We conceptually divide this research topic into four broad sections, which focus on the nature and attributes of picture and story books, what children learn from picture and story books, the interactions children experience during shared reading, and potential applications of research into shared reading, respectively.

## Attributes of picture and story books

The first section of this research topic focuses on the nature and structural attributes of picture and story books that influence the benefits of shared book reading. Three papers report empirical studies exploring how changes in story book attributes influence adult interaction style and child recall of story content (Greenhoot et al., 2014; Nyhout and O'Neill, 2014; Read, 2014). Greenhoot et al. (2014) examine the effect of storybook illustrations. Specifically, they gave parents either illustrated or non-illustrated stories to read with their 3–4-year-old children. Illustrated stories lead to more verbal and non-verbal exchanges between parents and children during shared reading and better recall of the story events by children. Nyhout and O'Neill (2014) explore the impact of narrative structure on book reading style. Parents read picture books to their 21-month-old children that either included contextual illustrations (narrative) or decontextualized illustrations (non-narrative). Although the two types of books elicited the same number of natural facts about animals overall, mothers reading narrative books provided more story-specific statements about the animals while mothers reading non-narrative books provided more labels and physical descriptions of animals. Finally, Read (2014) examined the effect of rhyme on children's learning of names for novel monsters. Two- to four-year-old children heard stories where either monster names did not rhyme with a descriptive feature, or where the name occurred before the feature (non-predictive rhyme) or after the feature (predictive rhyme). Children identified significantly more monsters by name if the names followed a predictive rhyme. Together, this sub-collection of papers demonstrates the dramatic influence picture and storybook attributes can have on parent reading behavior and children's learning.

The next two papers present cross-cultural comparisons of the messages conveyed by storybooks (Suprawati et al., 2014; Vander Wege et al., 2014). Suprawati et al. (2014) compared the nature of the challenges faced by protagonists in story books published in Indonesia, Japan and the United States, along with how these challenges were resolved. Japanese stories contained the largest number of challenges, and a greater proportion of challenges that were resolved by the protagonist alone compared to American stories. In a similar vein, Vander Wege et al. (2014) coded the illustrations of books published in Romania, Turkey and the United States for the depiction of emotional expressions. As predicted by cultural norms, American books showed more intense emotional reactions—especially negative expressions. These studies reveal that story books reflect (and may also teach children about) the values of one's cultural group.

The final two papers in this section consider the methods used by researchers to establish which features of story books best support learning. Wagner (2013) provides an opinion piece arguing for the need of a quantitative database of the content of children's books. She explains how such a database could be used to support theoretical claims about the content of picture books and to identify testable hypotheses about the features that help children learn to retell stories. A second methodological paper, by Burris and Brown (2014), reviews the external validity of narrative comprehension assessments, drawing particular attention to research with children from low-SES and minority populations. They argue that researchers should test comprehension using real-time, on-line assessments during the story reading (e.g., think-aloud protocols, probe questions), as well as off-line assessments of children's comprehension (e.g., free recall, cued recall and story retelling).

## Learning from picture and story books

The next section in the series focuses on what children learn from storybooks. Three papers focus specifically on word learning (Houston-Price et al., 2014; Khu et al., 2014; Williams and Horst, 2014) and a further six focus on how shared book reading supports developments in children's understanding of people (Abad and Pruden, 2013; Golos and Moses, 2013), animals (Ganea et al., 2014; Waxman et al., 2014), food (Heath et al., 2014), and even geometry concepts (Flevares and Schiff, 2014). Khu et al. (2014) demonstrate that teaching 21-month-old toddlers the name of a novel object through a picture book facilitates their ability to learn objects' non-obvious properties (e.g., lighting-up with applied pressure; introduced through a second picture book). Houston-Price et al. (2014) found that both 4- and 6-year-olds were able to provide accurate definitions of new words introduced in stories, but that only the older group formed lexical representations that enabled them to make correct grammaticality judgments about these words. By reading stories immediately before nap time, Williams and Horst (2014) were able to explore the added benefit of sleep on word learning from story books in a preschool sample. Together, these papers add to the literature demonstrating that children acquire new vocabulary knowledge through picture and storybooks (see also Read, 2014) and further elucidate the extent and depth of the knowledge gained.

The next two papers present opinions on how story book characters can help change children's stereotypes. First, Abad and Pruden (2013) synthesize what we know about the influence of story books whose characters engage in atypical gender behavior on children's subsequent play. The authors argue that story books provide a practical (and inexpensive) method for influencing gender stereotypes in a positive way. Golos and Moses (2013) take a similar approach to examining children's perceptions of deaf characters. In addition to reviewing how story books can help present the Deaf community positively, Golos and Moses make recommendations about the quality of the story line required if story books are to captivate and engage children.

The next pair of papers examines the effects of anthropomorphism in story books (Ganea et al., 2014; Waxman et al., 2014). In Ganea et al. (2014), 3–5-year-olds were read books with either anthropomorphic or realistic illustrations and either anthropomorphized or factual language. Both anthropomorphic illustrations and language lead to lower levels of learning, especially for the youngest children. In Waxman et al. (2014), 5-year-old children listened to a book about bears before completing a reasoning task. Children who read a book depicting bears scientifically (e.g., Animal Encyclopedia) generalized properties from one animal to another in the reasoning task (a biological perspective), while those who read a book depicting bears anthropomorphically (e.g., The Berenstain Bears) did not. Note, the stories used by Nyhout and O'Neill (2014) and Greenhoot et al. (2014) also included animals.

The final pair of papers in this section branch out to explore how story books can be used to change children's perceptions of food and mathematics (Heath et al., 2014). Heath et al. (2014) report that looking at picture books about an unfamiliar vegetable with toddlers encourages them to eat the vegetable when it is later offered at a mealtime. The effect was largest for foods that were unfamiliar to children before they saw the books. Heath et al. suggest that picture books might help more broadly to familiarize children with situations that they might otherwise reject. Flevares and Schiff (2014) undertake a chronological review of the evolution of different perspectives on the role of books in supporting school-aged children's learning of mathematical concepts, such as plane geometry. They explore the benefits of using picture-based literature for children's learning of and motivation to engage with mathematics concepts, and for the training of teachers in the delivery of these concepts.

## Interactions between readers and reading material

The third section in the series focuses on how children and parents interact with the story and each other during shared reading situations. Two papers in this section focus on the reading behaviors of middle-class African American (Harris and Rothlein, 2014) and Japanese (Murase, 2014) mothers. Harris and Rothlein (2014) found large individual differences in mothers' reading styles. However, the most common narrative-eliciting strategies included questions about the characters and refocusing statements (e.g., directing the child to look back at the illustration). Murase's (2014) 7-month longitudinal study of maternal reading behavior found that mothers initially focus on providing information to children and that they display more requests for information over time. The number of information-seeking requests by mothers was positively correlated with children's productive vocabularies. In the next paper, Kucirkova (2013) reviews how children interact with iPad books, which she suggests offer a useful tool for examining how children engage with stories. Kucirkova also highlights the need for researchers to acknowledge the learning opportunities provided by children's increasing experiences with digital media.

## Interventions using story books

The final section of the series takes a more applied angle. Two papers (Adlof et al., 2014; Tsunemi et al., 2014) present preliminary data from interventions using story books that show promise in helping children improve key skills. Adlof et al. (2014) confirmed the feasibility of a new intervention for low-SES children: structured narrative retell instruction (SNRI). In a group intervention, clinicians asked children questions about each component of the narrative (e.g., the characters) after each reading. Children who completed the intervention showed improvements in narrative macrostructure (including the total number and diversity of the words they used and their mean length of utterance) and overall vocabulary scores. Tsunemi et al.'s (2014) intervention aimed to help school-aged children with autism improve their social perspective-taking skills. Parents read narrative books to their children for almost a week and asked questions about the mental states of the characters after each reading. Children in the intervention group improved in their ability to take second- and third-person perspectives in a social perspective-taking task. Together, this pair of feasibility studies suggests that narrative storybooks provide a suitable medium for a range of interventions to support children's development.

## Conclusions

The goal of this Research Topic was to foster an interdisciplinary exchange of the methods that have been used to uncover how and what young children learn from books and the knowledge that this work has revealed. The final collection of articles has exceeded our expectations in regard to its breadth of offering, including work by researchers from fields comprising communication science, education, linguistics, psychology and speech and language disorders. The volume provides an eclectic but complementary overview of the current state of research on the status of picture and story books in young children's development. Our reading of this literature is that books are a powerful and somewhat unharnessed resource that could be employed to a much greater extent to help children to engage with and make sense of the world around them.

## Author contribution

Both JH and CH wrote the editorial and determined the groups of articles into the four categories presented in the editorial.

### Conflict of interest statement

The authors declare that the research was conducted in the absence of any commercial or financial relationships that could be construed as a potential conflict of interest.
